# Ocular manifestations and biomarkers of Gulf War Illness in US veterans

**DOI:** 10.1038/s41598-021-86061-0

**Published:** 2021-03-22

**Authors:** Brandon S. Baksh, Kristen L. Zayan, Raquel Goldhardt, Elizabeth R. Felix, Nancy Klimas, Anat Galor

**Affiliations:** 1 Ophthalmology, Miami Veterans Affairs Medical Center, Miami, FL USA; 2 University of Miami Miller School of Medicine, Miami, FL USA; 3 Bascom Palmer Eye Institute, University of Miami, 900 NW 17th Street, Miami, FL 33136 USA; 4 Research Services, Miami Veterans Affairs Medical Center, Miami, FL USA; 5 Physical Medicine and Rehabilitation, University of Miami, Miami, FL USA; 6 Nova Southeastern University, Ft Lauderdale, FL USA

**Keywords:** Optical imaging, Biomarkers

## Abstract

Gulf War Illness (GWI) is a multisystem disease with variable presentations, making diagnosis difficult. Non-invasive biomarkers would aid in disease diagnosis. We hypothesized that the eye could serve as a biomarker for GWI. We performed a retrospective case–control study using a sample of 1246 patients seen during a 5-month period in an optometry clinic. We identified veterans who were active duty during the Gulf War Era and either had a questionnaire-based diagnosis of GWI (cases) or did not (controls). Medical records were reviewed for eye and medical co-morbidities, medication use, and retinal macular and nerve fiber layer (NFL) thicknesses based on optical coherence tomography (OCT) images. Compared to controls (n = 85), individuals with GWI (n = 60) had a higher frequency of dry eye symptoms (50% vs 32.9%, p = 0.039). Multivariable analysis revealed average retinal NFL thickness (odds ratio; OR = 0.95), cup-to-disc ratio (OR = 0.005), age (OR = 0.82), and PTSD (OR = 20.5) were predictors of a GWI diagnosis. We conclude that GWI is associated with dry eye symptoms and RNFL thinning may serve as a biomarker for disease.

## Introduction

On return from the 1990 to 1991 Gulf War, about 200,000 veterans reported a wide range of symptoms that have been categorized as Gulf War Illness (GWI)^1^. GWI covers a wide range of symptoms including (1) fatigue (2) mood and cognition disorders and (3) musculoskeletal disorders. The pathophysiology of GWI is believed to involve central nervous system (CNS) dysfunction manifesting in multiple systems. Studies have examined CNS abnormalities in GWI. In a study of 96 veterans with GWI, functional magnetic resonance imaging (fMRI) showed significant decreases in the pre-frontal cortex and white-matter activity during high-demand working memory tasks compared to 44 matched controls^2^. These neurological changes have been linked to chemical exposure while in theater, including pesticides. A study of 7,971 United Kingdom Gulf War veterans (GWV) with GWI symptoms revealed a positive correlation between neurological symptoms and days handling pesticides, r = 0.08, p < 0.001^3^. Taken together, these data suggest GWI involves nervous system alterations in response to chemical exposures that have widespread biological effects.

Several age-related diseases have been found to be more common in GWI veterans compared to Gulf War Era (GWE) veterans not deployed to the Gulf War. These diseases include hypertension, coronary heart disease, and chronic obstructive pulmonary disease^4^. There is a paucity of data, however, on the frequency of age-related eye diseases in GWI, even though there is an increased frequency of blurry vision and photophobia in GWV compared to non-GWV^1^. Thus veterans with GWI may be at increased risk for age-related eye disease and this association should be explored. Furthermore, GWI may specifically be at risk for dry eye (DE) given the overlap in symptom profile between GWI, fibromyalgia, and myalgic encephalomyelitis/chronic fatigue syndrome (ME/CFS), the latter two of which have been associated with DE symptoms^5, 6^. Given these data, we hypothesized that individuals with GWI have a higher frequency of age-related eye diseases, including DE symptoms, compared to GWE veterans that do not meet GWI diagnostic criteria.

Beyond the frequency of overt disease, there is a need to identify sub-clinical biomarkers of GWI as diagnosis is difficult given varied presentations. A potential modality to identify sub-clinical disease is OCT, which has been employed to diagnose and monitor disease progression in Multiple Sclerosis (MS), Parkinson’s disease (PD), and Alzheimer’s disease (AD)^7–10^. For example, OCT parameters, such as retinal nerve fiber layer (RNFL) and ganglion cell layer (GCL) thickness correlated with changes in clinical status^11, 12^, visual acuity, and disability in MS^13–15^. Based on findings in other neurodegenerative diseases, we hypothesized that individuals with GWI, but without overt retinal and optic nerve pathology, would have differences in OCT measures compared to GWE veterans without a GWI diagnosis. To evaluate this hypothesis, we performed a retrospective case–control study.

## Materials and methods

### Study population and Gulf War Illness diagnosis

**Figure 1:**
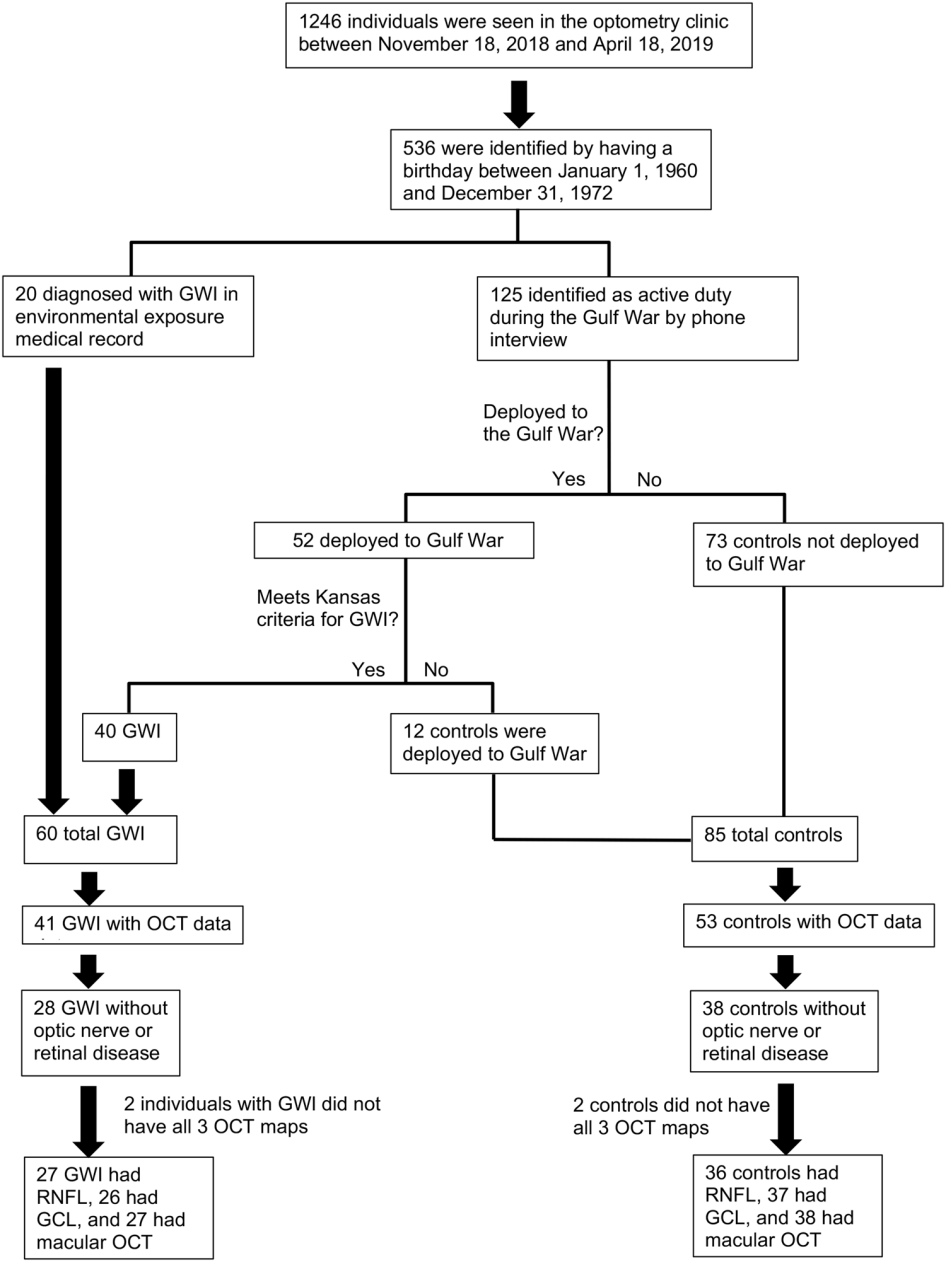
Flow chart of identification of veterans with Gulf War Illness. Of note, 2 of 28 individuals with GWI and 2 of 38 controls did not have all 3 OCT maps (RNFL, GCL-IPL, macula). RNFL images were available for 27 GWI and 36 controls, GCL for 26 GWI and 37 controls, and macula for 27 GWI and 38 controls. OCT = optical coherence tomography; GWI = Gulf War Illness. Figure was created using Microsoft Word for Mac (version 16.16.15, https://www.microsoft.com/en-us/microsoft-365/word).

The study population consisted of 1246 patients who were seen between November 18, 2018, and April 18, 2019, in the optometry clinic at the Miami Veterans Affairs Hospital (VA). Individuals were split into two groups: those with a diagnosis of GWI and those who served during the GWE who did not meet the criteria for GWI (controls) (Fig. 1). To identify all potential GWE veterans, we contacted 536 individuals seen in the optometry clinic during the relevant date range with a birthday between January 1, 1960, and December 31, 1972. Patients were diagnosed with GWI if they were deployed to the Gulf War and met the Kansas criteria via clinic or phone interview^1^. The Kansas criteria requires: symptoms started during or after deployment and were present in the year prior to assessment, and one severe or two moderate symptoms in at least three of six domains, including (1) fatigue, (2) pain, (3) neurologic and mood, (4) gastrointestinal, (5) respiratory, and (6) skin^1^. Veterans were included in the control group if they were active duty and deployed to the Gulf War, but did not meet Kansas criteria, or were active duty, but not deployed to the Gulf War.

Individuals with GWI were further sub-typed based on reported symptoms. Specific symptom clusters have been found to be useful when grouping GWI veterans^16^. In our study, we identified individuals with “severely impaired cognition” syndrome if they had at least 5 out of 6 of the following symptoms: problems with memory, feelings of irritability/angry outbursts, headaches, depression, difficulty concentrating, and trouble finding words when speaking.

The study was first approved by the Miami VA Institutional Review Board (IRB) as a quality assurance study. Approval was then obtained to link the questionnaire to clinical records. Informed consent was waived by the Miami VA IRB. The study was conducted in accordance with the principles of the Declaration of Helsinki and complied with the requirements of the United States Health Insurance Portability and Accountability Act.

### Data collected

Patient charts were retrospectively reviewed for demographics, co-morbidities, medications, and diagnoses of eye diseases including glaucoma, age-related macular degeneration, diabetic retinopathy, and DE symptoms or signs. DE symptoms were considered present if terms such as “dryness”, “irritation”, or “foreign body sensation” were listed as complaints in the clinical records. DE signs were considered present if any of the criteria were documented: fast tear break-up time (TBUT), positive fluorescein corneal staining, low tear lake, or Schirmer’s test < 5 mm wetting at 5 min.

### Imaging

Of 145 veterans identified via phone survey or medical record review as having served during the GWE, 94 individuals underwent OCT imaging (RNFL, GCL-inner plexiform layer (IPL), and macular maps) during their next routine clinic visit using a Cirrus HD-OCT (Carl Zeiss Meditec Inc, Dublin, California, USA). Of note, the 94 individuals with imaging were slightly older than the 51 who did not have OCT imaging available (51 ± 4.1 vs 52 ± 4.2, p = 0.013) but the remaining demographics were similar between the groups. OCT data from 28 individuals were subsequently excluded from the final analysis given overt retinal or optic nerve disease, including glaucoma, ocular hypertension retinopathy, retinal hemorrhage, diabetic retinopathy, or dry age-related macular degeneration (ARMD). Thus, 66 individuals with no diagnosis of retinal or optic nerve pathology and were included in the imaging analysis. Of note, 2 of 28 individuals with GWI and 2 of 38 controls did not have all 3 OCT maps (RNFL, GCL-IPL, macula). RNFL images were available for 27 GWI and 36 controls, GCL for 26 GWI and 37 controls, and macula for 27 GWI and 38 controls. For all analyses, the thinner RNFL, GCL, and macular value from either eye was used.

### Data analysis

Statistical analyses were performed using SPSS 24.0 (IBM Corp, Armonk, NY) statistical package. Descriptive statistics were used to summarize patient demographic and clinical information. Normality of the data was assessed using the Kolmogorov–Smirnov test. Differences in continuous variables between two groups were analyzed using the Student’s t-test or Mann–Whitney U test, as appropriate. Differences in continuous variables between more than two groups were analyzed using the Kruskal–Wallis H test. Differences in categorical data were compared using Chi-square or Fisher’s exact test, as appropriate^17^. Predictors of GWI were analyzed using forward stepwise binary logistic regression. All reported p-values are two-tailed and p < 0.05 was considered statistically significant. In this paper, we opted to give information on all variables being compared as opposed to correcting the p-value (e.g. Bonferroni) since the latter methodology has its own limitations^18^.

## Results

### Study population

During the above timeframe, 1246 veterans were seen in the optometry clinic. Of those, 145 served during the GWE, 60 met the criteria for GWI, and 85 served as controls. Twenty-eight GWI veterans were identified as having “severely impaired cognition.” Demographics were comparable between GWI veterans and controls (Table 1). Veterans with GWI had significantly higher frequencies of post-traumatic stress disorder (PTSD) (45% vs 20%, p = 0.001), chronic fatigue syndrome (13% vs 1%, p = 0.004), and fibromyalgia (18% vs 2%, p = 0.001) compared to controls. Of note, non-steroidal anti-inflammatory drug (NSAID) and naltrexone use were significantly more common in GWI vs controls (60% vs 42%, p = 0.036 and 15% vs. 0%, p < 0.001, respectively).

**Table 1:** Demographic and comorbidities of the study population.

	GWI (n = 60)	Control (n = 85)	P-value
**Demographics**			
Age (years)	52.1 ± 4.78 (45–71)	52.7 ± 3.84 (46–60)	0.39
Male gender	85% (51)	82% (70)	0.67
White race	41% (25)	37% (32)	0.81^a^
Hispanic ethnicity	26%(16)	20% (17)	0.42
**Non-ocular comorbidities**			
Diabetes	33% (20)	24% (21)	0.26
Hypertension	43% (26)	50% (43)	0.39
Hypercholesterolemia	51%(31)	49% (42)	0.92^a^
PTSD	45% (27)	20% (17)	0.001*
Depression	38% (23)	37% (32)	0.93
Arthritis	20% (12)	8% (7)	0.039*
Sleep apnea	56% (34)	47% (40)	0.25
Chronic fatigue syndrome	13% (8)	1% (1)	0.004^a^*
Fibromyalgia	18% (11)	2% (2)	0.001*
**Ocular comorbidities**			
Dry eye**	50% (30)	34% (29)	0.06
Symptoms	50% (30)	33% (28)	0.039*
Signs	23% (14)	18% (16)	0.51
Ocular hypertension	5% (3)	10% (9)	0.36^a^
Glaucoma	15% (9)	17% (15)	0.67
Cataract	11% (7)	7% (6)	0.34
Diabetic retinopathy	7% (4)	4% (3)	0.45^a^
Dry ARMD	3% (2)	0% (0)	0.17^a^
Wet ARMD	0% (0)	0% (0)	
Retinal hemorrhage	1% (1)	1% (1)	1.00^a^
Vitreous degeneration	3% (2)	4% (4)	1.00^a^
Keratoconus	5% (3)	2% (2)	0.65^a^
Any eye disease	73% (44)	61% (52)	0.13

Continuous variables are expressed as mean ± standard deviation (minimum–maximum). Categorical variables are expressed as percent (n). Mann–Whitney U test was used for all continuous variables. Pearson Chi Square was used for all categorical variables unless otherwise noted.

*GWI* Gulf War Illness, *Control* Individuals who served in 1990–91 who do not meet criteria for GWI, *ARMD* age-related macular degeneration, *SD* standard deviation, *n* number in group, *PTSD* post-traumatic stress disorder.

*Statistically significant difference at a p-value < 0.05 between GWI and control.

**Symptoms or signs.

^a^Fisher’s Exact Test.

### Frequency of eye diseases in the populations

Overall, individuals with GWI had a similar frequency of any eye disease, 73% vs 61%, p = 0.13. DE symptoms were significantly more common in GWI compared to controls, 50% vs 33%, p = 0.04. The GWI group tended to have higher frequencies of diabetic retinopathy (7% vs 4%, p = 0.45), and dry ARMD (3% vs 0%, p = 0.17), compared to controls, but the results were not significant with low frequencies in both groups. Compared to controls, GWI veterans with “severely impaired cognition” had significantly higher frequencies of both DE symptoms (61% vs 33%, p = 0.009) and signs (39% vs 19%, p = 0.028).

### Optical coherence tomography as a potential biomarker of Gulf War Illness

**Figure 2:**
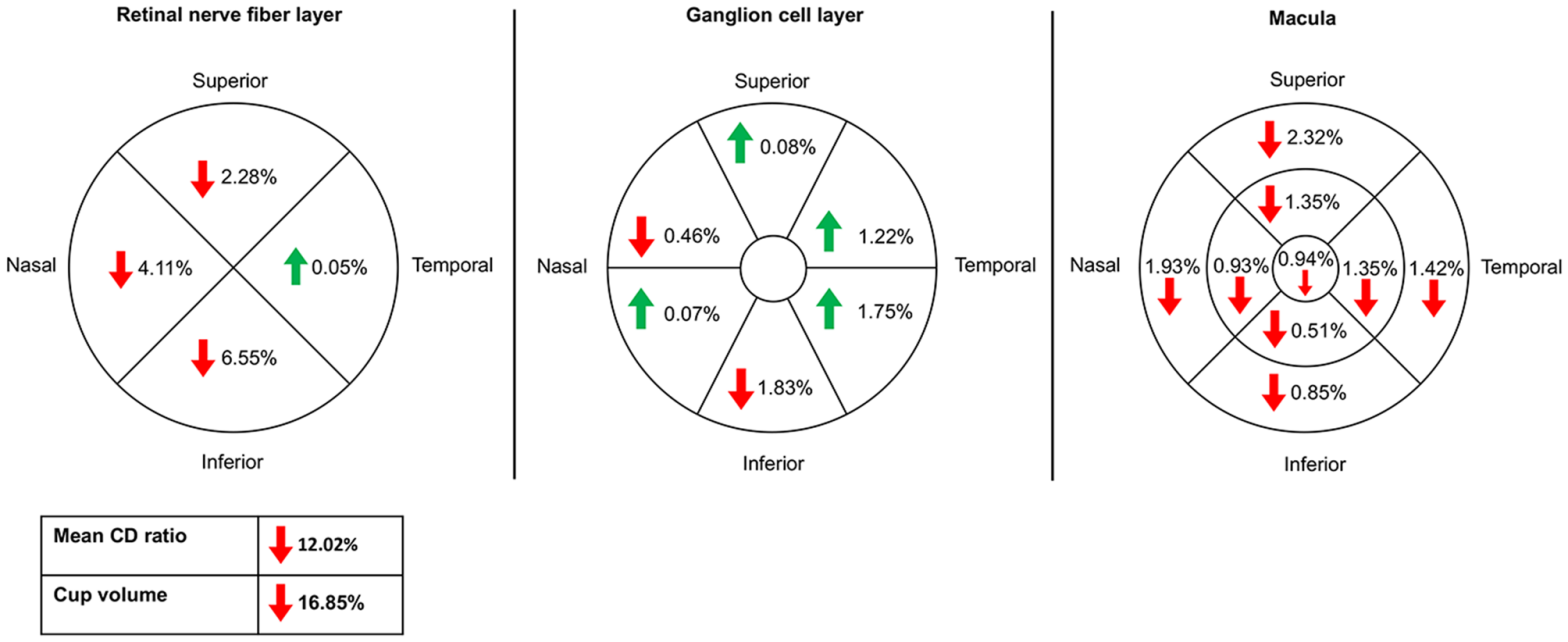
Percent change in optical coherence tomography measurements in Gulf War Illness. Percent change in optical coherence tomography (OCT) measurements Gulf War Illness (GWI) compared to controls. RNFL: n = 27 GWI and 36 controls. GCL: n = 26 GWI and 37 controls. Macula: n = 27 GWI and 38 controls. indicates a decreased percent change for GWI compared to controls. indicates an increased percent change. CD = cup-to-disc; OCT = optical coherence tomography. Figure was created using Microsoft PowerPoint for Mac (version 16.16.15, https://www.microsoft.com/en-us/microsoft-365/powerpoint).

Of the 94 individuals with available OCT images, 66 veterans (28 GWI and 38 controls) had no known optic nerve or retinal disease. Although not significant, almost all mean RNFL measurements were thinner in GWI compared to controls, with the largest difference seen in the inferior RNFL (109.33 μm ± 26.20 vs 117.00 μm ± 24.29, p = 0.13, a 6.6% decrease) (Supplementary Table S1 and Fig. 2). Similarly, all mean macular OCT measurements were thinner in veterans with GWI vs controls, with the largest decrease in the superior outer segment of the macula (271.00 μm ± 14.03 vs 277.45 μm ± 14.20, p = 0.12, a 2.32% decrease). Interestingly, almost all mean GCL parameters were thicker in the GWI group, with the largest increase in the inferotemporal GCL segment (78.65 μm ± 9.03 vs 77.29 μm ± 11.03, p = 0.245, a 1.75% increase). All other OCT measurements for GWI and controls are shown in Supplementary Table S1.

Further sub-grouping the population by severity of cognitive deficit, OCT data were available for 20 GWI veterans with “severely impaired cognition,” 7 GWI veterans without “severely impaired cognition,” and 38 controls. Of note, one individual with GWI was not included in this sub-analysis as questionnaire sub-score data was not available. Since OCT data for these groups were non-parametric, the Kruskal–Wallis H test was used to assess statistical differences between groups. GWI veterans with “severely impaired cognition” had significantly thinner inferior GCL (72.5 μm ± 12.0 vs 82.66 μm ± 2.74, p = 0.004, a 12% decrease) and inferotemporal GCL (76.8 μm ± 9.4 vs 85.0 μm ± 3.22, p = 0.011, a 9.7% decrease) compared to GWI veterans without the syndrome. The findings were similarly pronounced when GWI veterans with “severely impaired cognition” syndrome were compared to controls without GWI. Interestingly, GWI veterans without “severely impaired cognition” had significantly thicker values in inferior (82.67 μm ± 2.73 vs 76.24 μm ± 9.66, p = 0.015, a 8.42% increase) and inferotemporal GCL (85.00 μm ± 3.22 vs 77.30 μm ± 11.03, p = 0.006, a 9.97% increase) compared to controls without GWI.

### Predictors of Gulf War Illness

**Figure 3:**
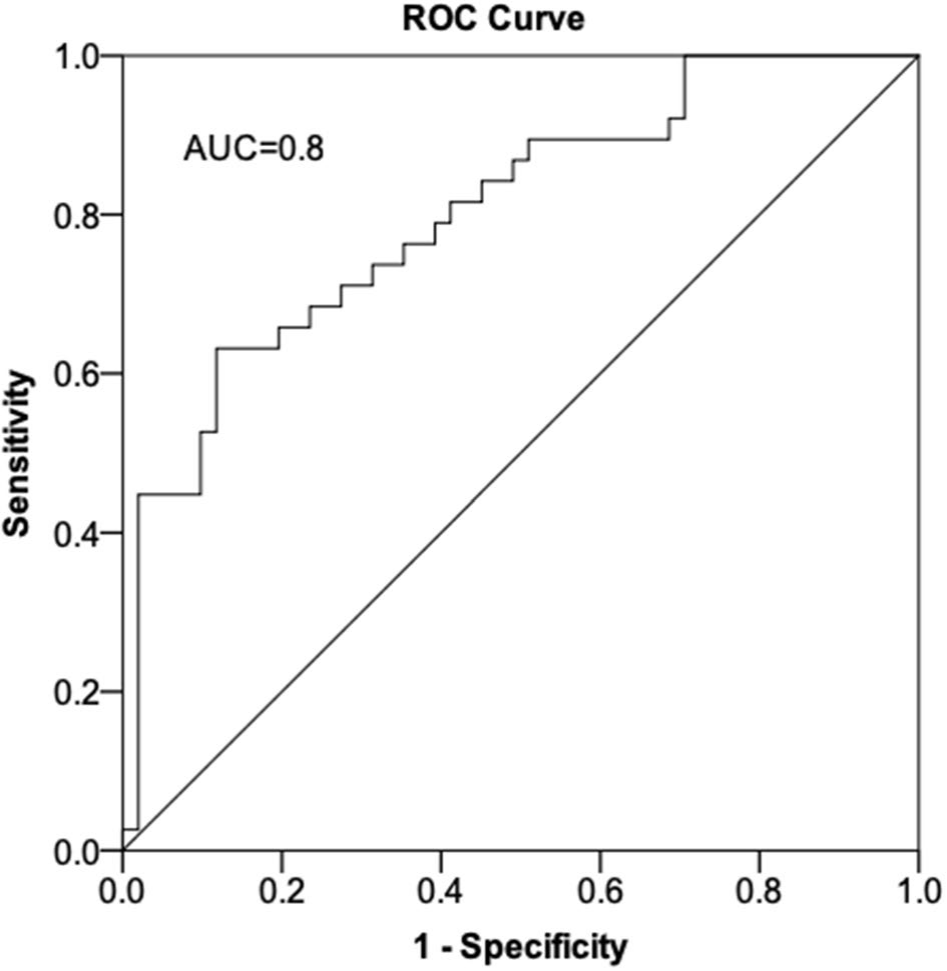
Receiver operating characteristic curve for predictors of Gulf War Illness. The curve is for a model using age, diagnosis of post-traumatic stress disorder (PTSD), overall retinal nerve fiber layer (RNFL) thickness, and cup-to-disc ratio as predictors. Forward stepwise binary logistic regression was used to develop the model. Receiver operating characteristic was used to test the ability of the model to predict a GWI diagnosis (yes/no). ROC = receiver operating characteristic. Figure was created using SPSS (version 24.0, https://www.ibm.com/products/spss-statistics).

To determine if specific demographics and OCT parameters could predict a diagnosis of GWI, we used all veterans with available OCT data to perform forward stepwise binary logistic regression with GWI (yes/no) as the dependent variable. Beyond OCT measures mentioned in the methods section, other metrics included as independent variables were demographics (age, gender, race, ethnicity), co-morbidities (PTSD and arthritis), NSAID use, and eye diseases (DE signs or symptoms, dry ARMD, diabetic retinopathy, ocular hypertension, retinal hemorrhage, and glaucoma). Fibromyalgia and chronic fatigue syndrome were not included in the model since their symptoms overlap with GWI. Naltrexone use was also excluded as it perfectly separated GWI from controls. Of note, population based differences in the 94 individuals included in the prediction analysis mirrored that of the entire population (n = 145), with individuals with GWI having a lower mean age, a higher frequency of PTSD and dry eye symptoms, and a trend toward thinner RNFL and macular thicknesses on OCT compared to controls. After confirming non-collinearity between predictors, the final model (Table 2) included age (odds ratio; OR = 0.82, 95% confidence interval; CI 0.70–0.96), PTSD (OR = 20.5, CI 95% 4.2–100.5), average RNFL thickness (OR = 0.95, CI 0.90–0.999), and average CD ratio (OR = 0.005, CI: 0.0–0.20). ROC analysis demonstrated an area under the curve (AUC) of 0.80 (95% CI 0.71–0.90, p < 0.001; Fig. 3) for this model in predicting a GWI diagnosis. The best cut-off value for the prediction model, as determined by Youden’s index (top left point on the ROC curve), was associated with a sensitivity of 76% and 60%. When excluding average RNFL thickness from the model, its predictive ability decreased (AUC = 0.68, 95% CI 0.59–0.77, p < 0.001).

**Table 2:** Results of forward stepwise binary logistic regression analysis for predictors of Gulf War Illness.

Predictor	β	S.E	Wald statistic	P value	OR	OR 95% CI
Age	− 0.20	0.08	6.03	0.014	0.82	0.70–0.96
PTSD	3.02	0.81	13.87	< 0.001	20.51	4.2–100.5
Mean RNFL thickness	− 0.05	0.03	4.03	0.045	0.95	0.90–0.999
Mean CD ratio	− 5.22	1.85	7.97	0.005	0.005	0.0–0.20

*PTSD* post-traumatic stress disorder, *RNFL* retinal nerve fiber layer, *CD* cup-to-disc, *OR* odds ratio, *CI* confidence interval, *S.E.* standard error for β.

## Discussion

In this study, we did not detect significant differences in the overall frequency of age-related eye diseases in individuals with GWI. However, dry eye symptoms were significantly more common in GWI compared to controls, which aligns with other diseases that have similar symptomology, such as fibromyalgia and chronic fatigue syndrome^5, 6^. There are many potential contributors to the noted association, including systemic inflammatory processes that lead to ocular surface inflammation and peripheral and/or central nerve abnormalities that lead to persistent symptoms of dryness^19, 20^.

Given the need for GWI biomarkers, we also compared retinal and optic nerve measures with OCT imaging. We found trends for macular OCT and RNFL thinning but GCL thickening in cases compared to controls. These differences became significant when GWI individuals with “severely impaired cognition” were compared to both individuals with GWI but without “severely impaired cognition” and controls. These data highlight the heterogeneous nature of GWI and suggest that different disease processes likely drive the clinical heterogeneity. Diagnostics tests are thus needed to detect disease but also to sub-type based on underlying pathophysiological mechanisms.

Similar to GWI, RNFL thinning has also been described in individuals with PD as compared to controls, with the temporal and nasal regions most affected^21^. In fact, the magnitude of inferior RNFL reduction in our GWI cohort (6.6%) is in the range of what has been reported in PD (6.2–15%^21^). This becomes relevant as studies have found other similarities between GWI and PD. In an MRI study of 293 Gulf War veterans compared to healthy controls, individuals with GWI had significantly more PD-like symptoms and reduced basal ganglia volumes (a common radiological feature of PD^22^)^23^. RNFL thinning has also been described in AD, with an overall mean reduction of 6.8–40% as compared to controls. Interestingly, the superior and temporal regions were most significantly affected in AD^21^, as compared to the nasal and inferior regions in GWI. OCT findings have also been reported in MS, with overall mean reductions in RNFL thickness of 7.2% and temporal reductions of 23% as compared to controls^24^. While the magnitude of overall RNFL reduction in GWI is smaller as compared to studies in PD, AD, and MS, these data highlight RNFL thinning as a marker of neurodegeneration with GWI showing similar trends.

Interestingly, in contrast to RNFL thinning, GCL values were thicker in GWI as compared to controls. This is the opposite of what has been described in other neurodegenerative diseases^21, 25^. We hypothesize that the discrepancy between GCL findings in GWI as compared to other neurodegenerative disorders are driven by competing mechanisms in GWI. While RNFL thinning can be an indicator of neurodegeneration, GCL thickening may indicate inflammation, with secondary edema, glial cell infiltration, and vascular changes^26^. Both processes have been implicated in GWI. A prospective MRI study found significant reductions in brainstem, cerebellar, and thalamus volumes in 17 GWI veterans compared to 23 controls, aligning with a neurodegenerative process^27^. On the other hand, neuroinflammation has also been described in GWI in the form of autoantibodies to neural and glial cell tissue, including calmodulin kinase II (CaMKII) and neurofilament triplet proteins (NFP), which are also found on retinal ganglion cells (RGC)^28–30^. Linking neurodegeneration and inflammation in GWI, one study found that increased serum concentrations of the inflammatory marker, soluble receptor II for tumor necrosis factor was significantly associated with reduced hippocampal volume in GWI veterans^31^. Thus, it is possible that individuals with diffuse inflammation on top of neurodegeneration may have RNFL thinning but GCL thickening while those with a more prominent neurodegeneration component have thinning in both layers^32, 33^. However, longitudinal studies are needed to evaluate our hypotheses.

In our final analysis, we explored whether specific OCT parameters could help discriminate between GWI and controls. We found that RNFL thickness, in conjunction with other parameters, predicted 80% of the variability in GWI risk. Similar regression analysis using minimum RNFL thickness and age predicted brain atrophy in patients with MS^34^. Other OCT parameters were also predictive of a GWI diagnosis including decreased average cup-to-disc (CD) ratio. This finding may be driven by mechanisms related to GWI, such as toxic or inflammatory changes, or may be due to an unrelated confounder, such as a higher frequency of individuals with physiologic cupping in the non-GWI group. Younger age also remained in the model as a predictor of GWI. This may simply reflect that of the population of individuals who were in service in 1990–1991, younger individuals were more likely to be deployed (a requirement for receiving a GWI diagnosis) than older individuals. Alternatively, younger age may be an unexplained contributor to GWI risk. Nevertheless, our data suggest that OCT has the potential to detect GWI and perhaps monitor disease progression. This is needed as GWI is a disease significant with morbidity and no disease-modifying therapies. Methods are thus needed to detect GWI early, identify GWI sub-types, and monitor for disease progression. Similar approaches have been investigated in other neuroinflammatory diseases, such as MS. In one study, OCT detected MS in the early stages of disease^35^, leading to early treatment which improved disease severity and morbidity^36^.

Our findings must be considered in light of the study limitations which included a retrospective design in a defined study population with a fixed sample size. As such, assessment of eye diseases was not performed in a standard method by individual clinicians. Furthermore, we used both phone and clinic-based interviews to define GWI. However, we used one of the two questionnaires (Kansas criteria) recommended by the Institute of Medicine and the United States Department of Defense in both settings^37^. Balancing the limitations are the strengths of this study which include the only study to our knowledge to evaluate ocular manifestations and non-invasive ocular biomarkers of GWI. Additional studies are thus needed in independent cohorts to replicate our findings and examine change over time, as has been done for other neurodegenerative diseases^38^. In fact, our plan is to further explore and validate the predictive markers discussed in this manuscript in a novel population. Despite these limitations, our findings open the possibility of studying OCT as biomarkers of GWI, which is greatly needed as GWI is a disease with high morbidity but with no therapeutic interventions.

## Supplementary information

Supplementary table S1.

## Data Availability

The datasets generated during and/or analyzed during the current study are not publicly available due to lack of permission from Veterans Health Administration to share data.
