# Supplementary material for: Ocular manifestations and biomarkers of Gulf War Illness in US veterans
Source: Sci Rep. 2021 Mar 22;11:6548. doi: 10.1038/s41598-021-86061-0 (PMC7985482; doi:10.1038/s41598-021-86061-0)
Supplement: Supplementary file 1 — Supplementary table S1. [file 41598_2021_86061_MOESM1_ESM.docx]

**Title**: Ocular manifestations and biomarkers of Gulf War Illness in US veterans

**Authors:** Brandon S Baksh^1,2^, Kristen Zayan^1,2^, Raquel Goldhardt^1,3^, Elizabeth Felix^4,5^, Nancy Klimas^4,6^, Anat Galor^1,3^*

**Affiliations:**

^1^Ophthalmology, Miami Veterans Affairs Medical Center, Miami, Florida, USA

^2^University of Miami Miller School of Medicine, Miami, Florida, USA

^3^Bascom Palmer Eye Institute, University of Miami, Miami, Florida, USA

^4^Research services, Miami Veterans Affairs Medical Center, Miami, Florida, USA

^5^Physical medicine and rehabilitation, University of Miami, Miami, Florida, USA

^6^Nova Southeastern University, Ft Lauderdale, Florida, USA

*****Corresponding author:

Name: Anat Galor

Address: 900 NW 17^th^ Street, Miami, Florida, 33136

Telephone: 305-326-6000

Email: agalor@med.miami.edu

Fax: 305-575-3312

| **OCT measurement** | **GWI (n=27)** | **Control (n=36)** | **P-value** |
| --- | --- | --- | --- |
| **Retinal nerve fiber layer (RNFL) imaging*** |  |  |  |
| Average NFL thickness (μm) | 88.26 ± 9.62 (70-106) | 91.86 ± 9.50 (67-106) | 0.14 |
| Superior NFL thickness (μm) | 110.67 ± 17.35 (64-139) | 113.25 ± 13.66 (85-137) | 0.52 |
| Nasal NFL thickness (μm) | 67.26 ± 8.79 (46-86) | 70.14 ± 10.33 (53-96) | 0.45 |
| Inferior NFL thickness (μm) | 109.33 ± 26.20 (40-79) | 117.00 ± 24.29 (31-86) | 0.13 |
| Temporal NFL thickness (μm) | 58.11 ± 10.90 (40-79) | 58.08 ± 10.03 (31-86) | 0.90 |
| Rim area (mm^2^) | 1.29 ± 0.25 (0.92-1.9) | 1.26 ± 0.22 (0.74-1.7) | 0.80 |
| Disc area (mm^2^) | 1.76 ± 0.27 (1.3-2.4) | 1.80 ± 0.29 (1.2-2.6) | 0.47 |
| CD ratio | 0.43 ± 0.19 (0.07-0.66) | 0.49 ± 0.15 (0.08-0.74) | 0.30 |
| Vertical CD ratio | 0.42 ± 0.19 (0.06-0.63) | 0.46 ± 0.14 (0.06-0.66) | 0.81 |
| Cup volume (mm^3^) | 0.14 ± 0.13 (0-0.44) | 0.17 ± 0.17 (0-0.84) | 0.49 |
| Any abnormality in NFL map | 66.7% (18) | 61.1% (22) | 0.65 |
|  | **GWI (n=26)** | **Control (n=37)** | **P-value** |
| **Ganglion cell layer (GCL) imaging*** |  |  |  |
| Superior GCL thickness (μm) | 78.85 ± 9.46 (48-92) | 78.78 ± 10.55 (33-94) | 0.89 |
| Superonasal GCL thickness (μm) | 78.77 ± 9.61 (48-92) | 79.14 ± 10.63 (35-95) | 0.99 |
| Inferonasal GCL thickness (μm) | 77.27 ± 9.77 (46-90) | 77.22 ± 10.06 (38-93) | 0.69 |
| Inferior GCL thickness (μm) | 74.85 ± 11.43 (37-86) | 76.24 ± 9.66 (34-93) | 0.88 |
| Inferotemporal GCL thickness (μm) | 78.65 ± 9.03 (45-89) | 77.29 ± 11.03 (29-96) | 0.25 |
| Superotemporal GCL thickness (μm) | 77.42 ± 8.78 (49-89) | 76.49 ± 10.69 (31-98) | 0.46 |
| Overall GCL + IPL thickness (μm) | 78.12 ± 8.34 (49-88) | 77.86 ± 10.09 (33-93) | 0.73 |
| Minimum GCL thickness (μm) | 72.92 ± 13.23 (29-85) | 73.76 ± 12.33 (19-90) | 0.81 |
| Any abnormality in ganglion cell layer map | 26.9% (7) | 33.3% (12) | 0.59 |
|  | **GWI (n=27)** | **Control (n=38)** | **P-value** |
| **Macula thickness imaging*** |  |  |  |
| Central macula thickness (μm) | 244.44 ± 25.82 (185-295) | 246.76 ± 22.06 (214-304) | 0.80 |
| **Inner Ring** |  |  |  |
| Superior macula thickness (μm) | 313.85 ± 16.53 (276-339) | 318.13 ± 16.03 (279-357) | 0.42 |
| Nasal macula thickness (μm) | 314.00 ± 16.73 (278-342) | 316.95 ± 19.92 (250-352) | 0.59 |
| Inferior macula thickness (μm) | 310.04 ± 16.32 (270-332) | 311.63 ± 20.33 (230-351) | 0.57 |
| Temporal macula thickness (μm) | 301.67 ± 15.67 (275-327) | 305.79 ± 16.37 (278-334) | 0.41 |
| **Outer Ring** |  |  |  |
| Superior macula thickness (μm) | 271.00 ± 14.03 (231-291) | 277.45 ± 14.20 (244-314) | 0.12 |
| Nasal macula thickness (μm) | 285.70 ± 17.54 (233-314) | 291.32 ± 16.41 (261-335) | 0.29 |
| Inferior macula thickness (μm) | 260.85 ± 12.99 (224-281) | 263.08 ± 14.52 (238-298) | 0.91 |
| Temporal macula thickness (μm) | 257.00 ± 9.40 (234-271) | 260.71 ± 13.40 (241-292) | 0.41 |
| Any abnormality in macula map | 48.1% (13) | 50.0% (19) | 0.88 |

**Supplementary Table S1:** Optical coherence tomography thickness parameters in cases vs controls**.** Continuous variables are expressed as mean ± standard deviation (minimum-maximum). Categorical variables are expressed as percent (n). Mann-Whitney U test was used for all continuous variables. Pearson Chi square was used for all categorical variables. No significant differences were found. *Thinner value from either eye selected for each individual; GWI=Gulf War Illness; Control=Individuals who served in 1990-91 who do not meet criteria for GWI; SD=standard deviation; n=number in group; NFL=nerve fiber layer; CD=cup-to-disc.
